# Prognostic differences between persistent HFrEF and HFrecEF following acute myocardial infarction

**DOI:** 10.3389/fcvm.2025.1597947

**Published:** 2025-07-17

**Authors:** Jeong Yoon Jang, Jae Myoung Lee, Yujin Shin, Yong-Lee Kim, Gain Yu, Jae Seok Bae, Yun-Ho Cho, Choong Hwan Kwak, Min Gyu Kang, Kye-Hwan Kim, Jeong Rang Park, Jin-Yong Hwang, Young-Hoon Jeong, Jong-Hwa Ahn

**Affiliations:** ^1^ Division of Cardiology, Department of Internal Medicine, Gyeongsang National University School of Medicine and Gyeongsang National University Changwon Hospital, Changwon, Republic of Korea; ^2^ Division of Cardiology, Department of Internal Medicine, Gyeongsang National University School of Medicine and Gyeongsang National University Hospital, Jinju, Republic of Korea; ^3^ Gwangmyeong and Department of Internal Medicine, CAU Thrombosis and Biomarker Center, Chung-Ang University Gwangmyeong Hospital, Chung-Ang University College of Medicine, Seoul, Republic of Korea

**Keywords:** AMI, HFREF, HFrecEF, prognosis, predictors

## Abstract

**Background:**

Acute myocardial infarction (AMI) often leads to heart failure with reduced ejection fraction (HFrEF), with some patients showing recovery of left ventricular ejection fraction (HFrecEF) over time. This study aimed to evaluate the prognostic differences between persistent HFrEF and HFrecEF.

**Methods:**

This prospective cohort study included AMI patients with reduced LVEF (<40%) at admission. LVEF was reassessed one month later to classify patients into persistent HFrEF (LVEF <40%) or HFrecEF, defined as follow-up LVEF >40% with an absolute increase of ≥10% from baseline, in accordance with recent consensus definitions. Outcomes included cardiovascular mortality and/or rehospitalization for heart failure. Predictors of LVEF recovery were also analyzed.

**Results:**

Of the 679 patients analyzed, 373 (55%) had persistent HFrEF, while 306 (45%) transitioned to HFrecEF. Patients with HFrecEF were younger, had fewer comorbidities, and were more likely to receive renin-angiotensin system (RAS) inhibitors and β-blockers.Cardiovascular mortality was significantly lower in the HFrecEF group (3.3% vs. 8.3%; adjusted HR 0.37, 95% CI: 0.18–0.77, *p* = 0.007), as was the rate of heart failure rehospitalization (6.2% vs. 10.2%; adjusted HR 0.60, 95% CI: 0.35–1.05, *p* = 0.074). Independent predictors of LVEF recovery included younger age, beta-blocker use, and RAS inhibitor use.

**Conclusion:**

This study emphasizes the critical role of transitioning from persistent HFrEF to HFrecEF in improving clinical outcomes for AMI patients. Tailored management approaches, combined with routine echocardiographic monitoring and adherence to optimal medical therapy, are essential for optimizing patient care and long-term prognosis.

## Introduction

Heart failure with reduced ejection fraction (HFrEF) is a common complication of acute myocardial infarction (AMI), driven by significant myocardial injury and subsequent ventricular remodeling (1, 2). While many patients experience persistent ventricular dysfunction, others achieve substantial recovery of left ventricular ejection fraction (LVEF), a condition termed heart failure with recovered ejection fraction (HFrecEF) (2). This phenomenon has garnered increasing clinical attention due to its implications for long-term outcomes and management strategies (3, 4).

Despite the improved prognosis associated with HFrecEF, the underlying mechanisms facilitating LVEF recovery remain poorly understood. Factors such as age, baseline LVEF, adherence to guideline-directed medical therapy (GDMT), and comorbidities have been proposed as potential contributors (2, 4, 5). Moreover, the prognostic disparities between patients with persistent HFrEF and HFrecEF highlight the need for a nuanced approach to post-AMI care.

This study aims to investigate the prognostic differences between persistent HFrEF and HFrecEF following AMI. By identifying predictors of LVEF recovery and examining associated clinical outcomes, we seek to inform strategies for optimizing management and improving long-term survival in this population.

## Methods

### Study design and population

This prospective, multicenter registry-based cohort study was conducted at Gyeongsang National University Changwon Hospital and Gyeongsang National University Hospital (Jinju), which share standardized clinical protocols and a unified electronic data management system (6) (NCT04650529). Consecutive patients with significant CAD who underwent PCI (Jinju and Changwon) between January 2010 and November 2020 were enrolled in this registry, which evaluated multiple vascular, hemostatic, and physiological parameters, if available.

This prospective cohort study included patients admitted for AMI, who had LVEF <40% at admission. Follow-up echocardiography was performed at 1 month to classify patients into persistent HFrEF (LVEF <40%) and HFrecEF, defined as follow-up LVEF >40% with an absolute increase of ≥10% from baseline, in accordance with recent consensus definitions (7).

### Data collection and outcomes

Clinical, laboratory, and echocardiographic data were collected using a standardized case report form by trained study coordinators at each center. All data were prospectively recorded based on a unified study protocol. Additional information was obtained from hospital electronic medical records or by contacting the principal investigators when necessary. Outcomes of interest included cardiovascular mortality and/or rehospitalization for heart failure, confirmed through medical records or telephone contact with patients or family members.

### Statistical analysis

Statistical analyses were conducted using SPSS version 26.0 (IBM Corp., Armonk, NY, USA). Categorical variables were compared using chi-square tests or Fisher's exact tests, as appropriate. Continuous variables were analyzed using independent t-tests or Mann–Whitney *U*-tests, based on data distribution. Survival analyses were performed using Kaplan–Meier methods with log-rank tests to compare survival curves. Multivariate Cox proportional hazards regression models were used to identify predictors of outcomes, adjusting for potential confounders such as age, sex, and comorbidities. To identify clinical predictors of LVEF recovery at 1-month follow-up, we first performed univariate logistic regression analyses using baseline demographic, clinical, and treatment variables. Variables with *p*-values < 0.05 in univariate analysis were considered candidates for multivariable modeling. A multivariable logistic regression model was constructed using backward stepwise selection to determine independent predictors of LVEF recovery (HFrecEF). Variables considered for inclusion in the model were based on clinical relevance and included age, sex, height, weight, hypertension, diabetes mellitus, current smoking, chronic kidney disease, LAD-PCI, complex PCI, and use of discharge medications (angiotensin blockade, beta-blockers, calcium channel blockers). The final model retained only variables that remained statistically significant (*p* < 0.05).

## Results

### Study flow

**Figure 1:**
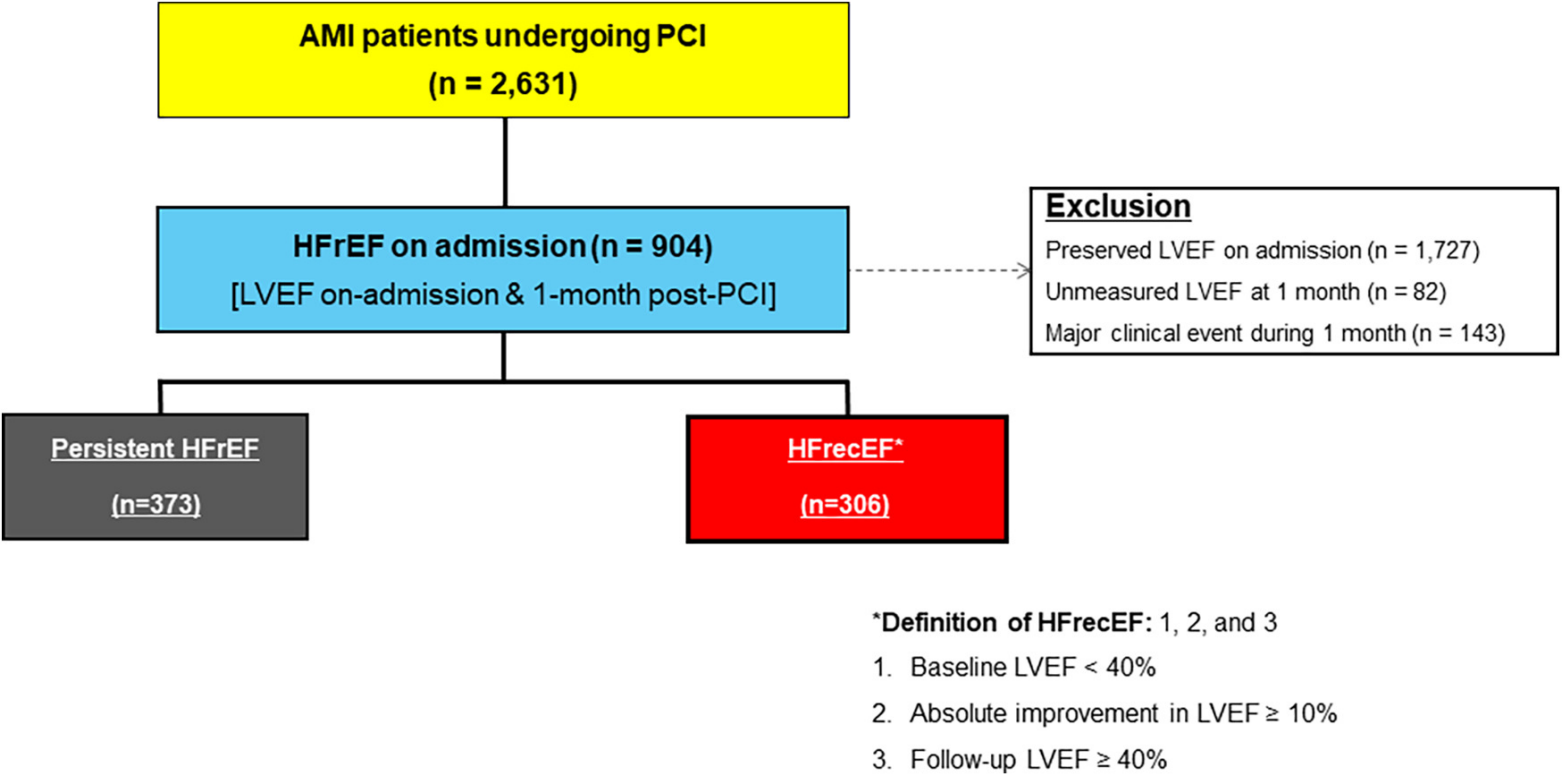
Study flow diagram. AMI, acute myocardial infarction; HFrecEF, heart failure with recovered ejection fraction; HFrEF, heart failure with reduced ejection fraction; PCI, percutaneous coronary intervention.

Figure 1 illustrates the study flow diagram. Of 2,631 AMI patients undergoing PCI, 904 had reduced LVEF at admission. After excluding those with preserved LVEF (1,727), unmeasured LVEF at 1 month (82), and major clinical events during the first month (143), 679 patients remained, classified into persistent HFrEF (373) and HFrecEF (306).

### Baseline characteristics

Patients with HFrecEF were younger (66.4 ± 12.5 years vs. 69.2 ± 12.8 years, *p* < 0.001) and had higher on-admission LVEF (34.1 ± 6.3% vs. 35.7 ± 4.8%, *p* < 0.001). Beta-blockers (85.9% vs. 77.2%, *p* = 0.013) and RAS inhibitor (85.0% vs. 74.8%, *p* < 0.001) were higher in they were more in HFrecEF group. At the 1-month follow-up, their LVEF further improved to 56.1 ± 4.7%, compared to 42.5 ± 5.6% in the persistent HFrEF group. HFrecEF patients also exhibited fewer comorbidities, including hypertension (41.8% vs. 55.5%, *p* < 0.001) and chronic kidney disease (21.6% vs. 27.9%, *p* < 0.001) (Table 1).

**Table 1:** Baseline characteristics of study groups.

Variables	Persistent HFrEF (*n* = 373)	HFrecEF (*n* = 306)	*P*-value
LV ejection fraction, %			
On-admission	34.1 ± 6.3	35.7 ± 4.8	<0.001
1-month follow-up	42.5 ± 5.6	56.1 ± 4.7	
Index presentation, *n* (%)			0.001
Non-ST-segment elevation MI	164 (44.0)	145 (47.4)	
ST-segment elevation MI	209 (56.0)	161 (52.6)	
Age, years	69.2 ± 12.8	66.4 ± 12.5	<0.001
Male, *n* (%)	250 (67.0)	214 (69.9)	0.005
Body mass index, kg/m²	23.4 ± 3.7	23.6 ± 3.7	<0.001
Previous history, *n* (%)			
Previous PCI	29 (7.8)	11 (3.6)	0.010
Previous stroke	28 (7.5)	23 (7.5)	0.371
Risk factor, *n* (%)			
Hypertension	207 (55.5)	128 (41.8)	<0.001
Diabetes mellitus	119 (31.9)	95 (31.0)	<0.001
Dyslipidemia	200 (53.6)	193 (63.1)	0.005
Smoking	149 (39.9)	130 (42.5)	0.034
Chronic kidney disease	104 (27.9)	66 (21.6)	<0.001
Anemia	113 (30.3)	75 (24.5)	<0.001
Laboratory measurements			
White blood cell, ×10^3^/mm^3^	11.4 ± 4.4	10.7 ± 3.5	<0.001
Hemoglobin, g/dl	13.1 ± 2.0	13.5 ± 2.2	<0.001
Platelet, ×10^3^/mm^3^	253.9 ± 79.0	258.2 ± 85.3	0.020
Glomerular filtration rate, ml/min/1.73m^2^	77.6 ± 34.2	83.5 ± 35.8	<0.001
Total cholesterol, mg/dl	186.2 ± 47.3	191.1 ± 48.2	0.036
HbA1c, %	6.48 ± 1.39	6.66 ± 1.37	0.002
Procedural characteristics			
Culprit lesion			<0.001
Left main coronary artery	10 (2.7)	9 (2.9)	
Left anterior descending artery	241 (64.6)	199 (65.0)	
Left circumflex artery	75 (20.1)	65 (21.2)	
Right coronary artery	115 (30.8)	109 (35.6)	
Multivessel disease, *n* (%)	211 (56.6)	158 (51.6)	0.001
Concomitant medications, *n* (%)			
Aspirin	370 (99.2)	304 (99.3)	0.927
P2Y12 receptor inhibition			0.007
Clopidogrel	302 (81.0)	256 (83.7)	
Prasugrel	13 (3.5)	8 (2.6)	
Ticagrelor	53 (14.2)	38 (12.4)	
Beta blocker	288 (77.2)	263 (85.9)	0.013
Angiotensin blockade	279 (74.8)	260 (85.0)	<0.001
Statin	356 (95.4)	297 (97.1)	0.114

Values are mean ± SD or *n* (%). MI, myocardial infarction; HFrecEF, heart failure with recovered ejection fraction; HFrEF, heart failure with reduced ejection fraction; HbA1c, hemoglobin A1c; PCI, percutaneous coronary intervention.

### Predictors of LVEF recovery

Logistic regression analysis identified younger age (OR 0.98, *p* = 0.022), beta-blocker use (OR 1.60, *p* = 0.039), and RAS inhibitor use (OR 1.66, *p* = 0.022) as independent predictors of LVEF recovery (Table 2).

**Table 2:** The independent predictors of recovered LV function after AMI in the logistic regression analyses.

Univariate			Multiple (Final model)		
Parameter	Odds Ratio	*p*	Odds Ratio	Standard Error	*p*
Age	0.99	0.091	0.98	0.01	*0*.*022*
Sex	0.88	0.644	–	–	–
Height	1.00	0.875	–	–	–
Body weight	1.00	0.666	–	–	–
Hypertension	0.62	0.009	–	–	–
Diabetes mellites	1.11	0.598	–	–	–
Current smoker	0.80	0.263	–	–	–
Chronic kidney disease	0.80	0.281	–	–	–
LAD PCI	0.85	0.366	–	–	–
Complex PCI	0.86	0.587	–	–	–
Angiotensin blockade	1.67	0.019	1.66	0.22	*0*.*022*
Beta blocker	1.64	0.030	1.60	0.23	*0*.*039*
Calcium channel blocker	0.68	0.500	–	–	–

LAD, left anterior descending artery; PCI, percutaneous coronary intervention.

### Clinical outcomes

**Figure 2:**
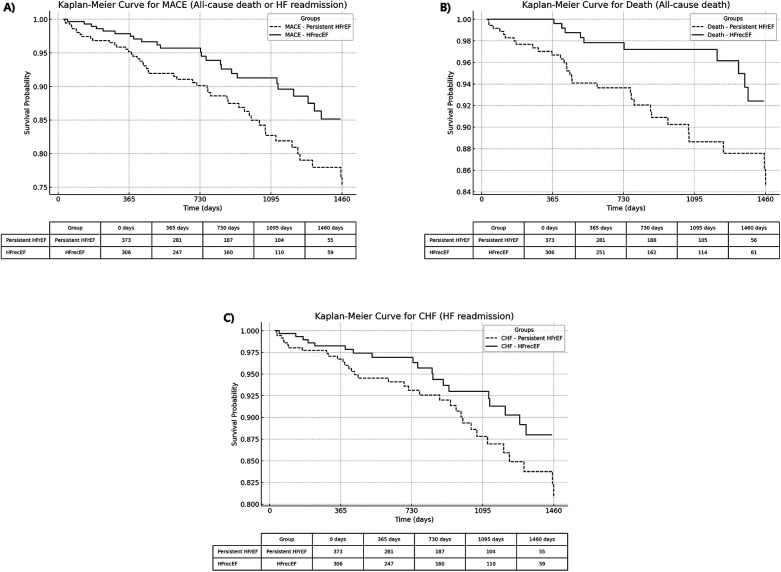
Kaplan–Meier curves comparing adverse events between HFrecEF and persistent HFrEF groups. **(A)** All-cause death or heart failure readmission; **(B)** All-cause death; **(C)** Heart failure readmission. Solid lines represent the HFrecEF group; dashed lines represent the persistent HFrEF group. HFrecEF, heart failure with recovered ejection fraction; HFrEF, heart failure with reduced ejection fraction.

Kaplan–Meier curves (Figure 2) demonstrate significantly better outcomes in the HFrecEF group. The composite outcome of all-cause death or heart failure readmission occurred in 7.8% of HFrecEF patients compared to 13.9% in the persistent HFrEF group (adjusted HR 0.55, 95% CI: 0.34–0.90, *p* = 0.018). HFrecEF patients also had lower rates of all-cause death (3.3% vs. 8.3%, adjusted HR 0.37, 95% CI: 0.18–0.77, *p* = 0.007). While HF readmission rates were lower in HFrecEF (6.2% vs. 10.2%), the adjusted model showed a trend towards significance (adjusted HR 0.60, 95% CI: 0.35–1.05, *p* = 0.074) (Table 3).

**Table 3:** Clinical outcomes according to recovered LVEF after AMI.

Events	Rates		Unadjusted model			Adjusted model*		
	Persistent HFrEF (*n* = 373)	HFrecEF (*n* = 306)	HR	95% CI	*P*-value	HR	95% CI	*P*-value
All-cause death or HF readmission	52 (13.9%)	24 (7.8%)	0.50	0.31–0.81	0.005	0.55	0.34–0.90	0.018
: All-cause death	31 (8.3%)	10 (3.3%)	0.35	0.17–0.71	0.004	0.37	0.18–0.77	0.007
: HF readmission	38 (10.2%)	19 (6.2%)	0.54	0.31–0.93	0.028	0.60	0.35–1.05	0.074

* Adjusted for index STEMI presentation, age, gender, BMI, smoking, DM, hypertension, dyslipidemia, CKD, multivessel disease.

Values are *n* (%). CI, confidence interval; HR, hazard ratio.

## Discussion

This study adds to the growing body of evidence on the prognostic significance of early LVEF recovery following AMI by comparing clinical outcomes between patients with persistent HFrEF and those with HFrecEF. Patients with HFrecEF demonstrated more favorable outcomes, including lower rates of cardiovascular mortality and heart failure rehospitalization. These findings support the clinical relevance of early post-AMI echocardiographic reassessment and may inform future strategies for risk stratification and individualized management across different heart failure phenotypes.The results highlight that the importance of adhering to guideline-directed medical therapy (GDMT) and serial assessment of left ventricular function by echocardiography can be used to predict the clinical outcome of patients underwent PCI due to ACS (8, 9).

Younger age and the use of beta-blockers and RAS inhibitors emerged as pivotal predictors of LVEF recovery, offering actionable insights into optimizing care for at-risk populations (2). This aligns with prior evidence suggesting that early initiation of GDMT, combined with consistent patient adherence, can significantly enhance outcomes in heart failure patients (8, 9).

Despite these advancements, persistent HFrEF remains a challenging phenotype, characterized by higher risks and poor outcomes. Comprehensive, individualized approaches combining pharmacological and non-pharmacological strategies are needed to address this group effectively. Device-based therapies, such as implantable cardioverter defibrillators (ICDs) or cardiac resynchronization therapy (CRT), may offer additional benefits for patients with severe ventricular dysfunction (7, 9). Moreover, lifestyle interventions, including dietary optimization and supervised exercise programs, could further improve functional status and quality of life (10, 11).

These findings underline the heterogeneity in heart failure phenotypes and the necessity for precision medicine in this domain. Future research should focus on exploring novel pharmacological agents, such as SGLT2 inhibitors, which have shown promise in recent trials for heart failure management (7, 9, 12). Furthermore, long-term studies investigating the durability of LVEF recovery and its impact on survival will be crucial for advancing care in this population. Younger age and the use of beta-blockers and RAS inhibitors emerged as pivotal predictors of LVEF recovery, offering actionable insights into optimizing care for at-risk populations. Although younger age was identified as a predictor of LVEF recovery, this could also underscore the importance of early detection, close follow-up, and the optimization of medical therapy in elderly patients, who may have more a limited potential for spontaneous recovery. Strategies such as comprehensive geriatric assessment, frailty screening, and enhanced support for medication adherence may contribute to improve outcomes in this population.

Nonetheless, a significant proportion of patients continue to experience persistent HFrEF, highlighting an unmet clinical need. Comprehensive, individualized approaches combining pharmacological and non-pharmacological strategies are needed to address this group effectively. These findings underline the heterogeneity in heart failure phenotypes and the necessity for precision medicine in this domain.

## Limitations

This study has certain limitations. First, as a single-cohort study, residual confounding cannot be ruled out. Factors such as infarct size, extent of coronary artery disease, prior revascularization history, adherence to medications, and socioeconomic variables were not fully captured in our dataset and may influence the observed associations*.* Second, the exclusion of patients with unmeasured LVEF or major clinical events may have resulted in a selection bias And due to the nature of our registry, written informed consent was obtained only from patients who survived the initial 1-month post-AMI period without major events, in accordance with IRB requirements. As a result, patients with early death or major complications were not enrolled, which may introduce selection bias and limit the generalizability of our findings. Third, our dataset lacked comprehensive information on the use of mineralocorticoid receptor antagonists (MRAs) and sodium-glucose co-transporter 2 (SGLT2) inhibitors. As a result, our analysis could not fully assess the implementation of contemporary guideline-directed medical therapy (GDMT), and the findings regarding pharmacologic treatment are primarily limited to RAS inhibitors and *β*-blockers. Fourth, interobserver variability in echocardiographic assessments could influence group classification.

Finally, although the study used a 1-month follow-up period in accordance with routine post-AMI clinical practice, we acknowledge that this time frame may not fully capture the extent or durability of left ventricular reverse remodeling, which can evolve over 3 to 6 months. Future studies incorporating serial echocardiographic evaluations over longer follow-up periods and multicenter validation are warranted to confirm and extend our findings.

## Conclusion

In conclusion, this study highlights the critical prognostic differences between persistent HFrEF and HFrecEF following AMI. The transition to HFrecEF is associated with significantly better outcomes, driven by predictors such as younger age and the use of beta-blockers and RAS inhibitors. These findings underscore the importance of regular echocardiographic monitoring and tailored therapeutic strategies to optimize recovery.

## Data Availability

The raw data supporting the conclusions of this article will be made available by the authors, without undue reservation.
